# Supplementary material for: Neuroinflammation-induced lymphangiogenesis near the cribriform plate contributes to drainage of CNS-derived antigens and immune cells
Source: Nat Commun. 2019 Jan 16;10:229. doi: 10.1038/s41467-018-08163-0 (PMC6335416; doi:10.1038/s41467-018-08163-0)
Supplement: Supplementary file 1 — Supplementary Information [file 41467_2018_8163_MOESM1_ESM.docx]

**Neuroinflammation-induced lymphangiogenesis near the cribriform plate contributes to drainage of CNS-derived antigens and immune cells.**

Hsu et al.

Corresponding Author:

Zsuzsanna Fabry

Email: [zfabry@wisc.edu](mailto:zfabry@wisc.edu)

Phone: (608) 265-8716

This work was supported by National Institutes of Health grants NS108497 and NS103506 (to Z.F.), HL128778 (to M.S.), AI101378 (to W.J.K.), the Neuroscience Training Program T32-GM007507 (to M.H. and A.R.), and the American Heart Association award 15PRE25500022 (to A.R.). The authors have no financial or personal conflict of interest.


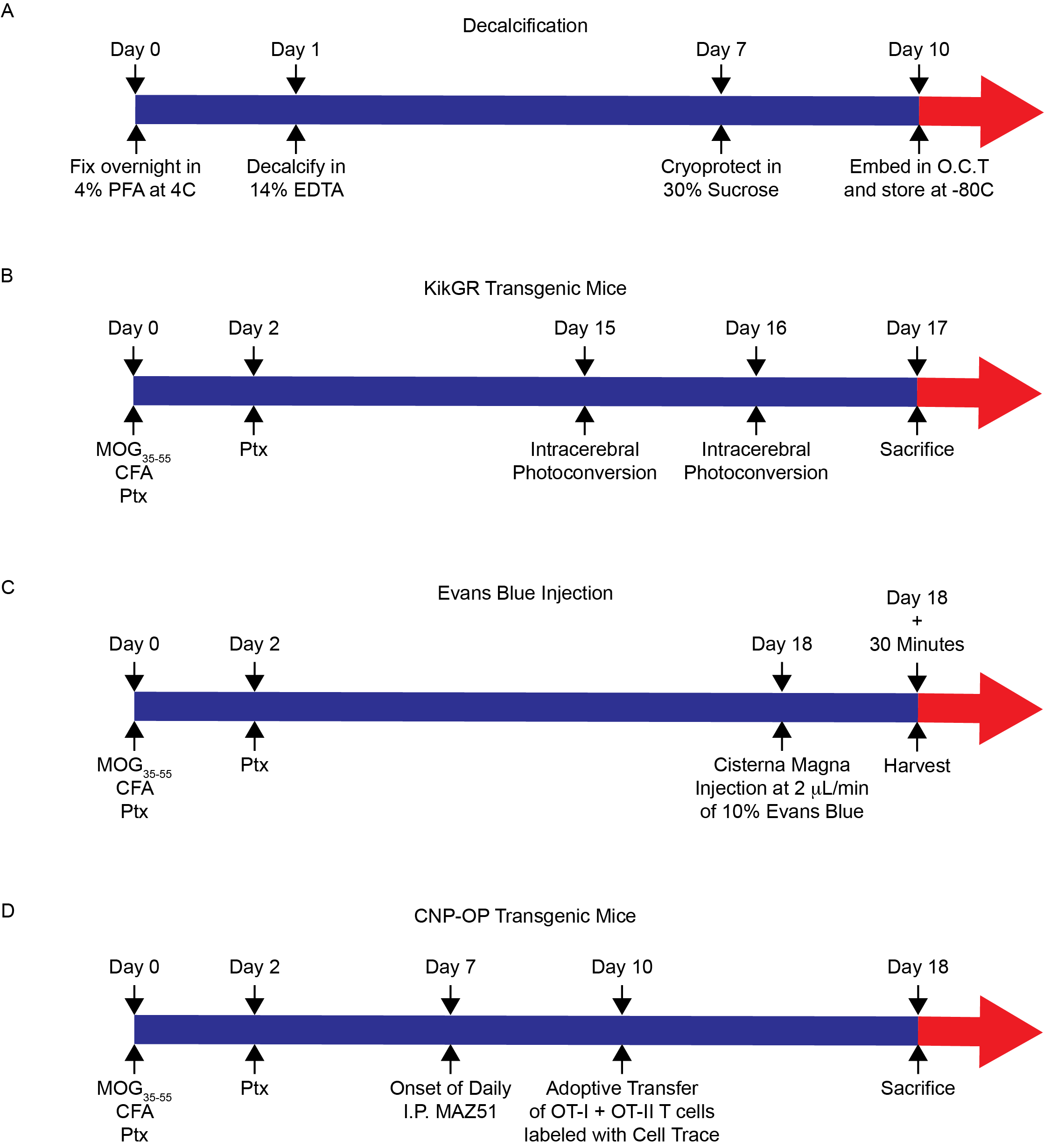


**Supplementary Figure 1: Summary of Experimental Designs**

**(A):** Experimental design for the decalcification of whole mouse heads. Mice were perfused with ice cold PBS followed by perfusion with 4% PFA. The whole heads were dissected and further fixed overnight in 4% PFA, followed by decalcification in 14% EDTA for 7 days. The heads were then cryoprotected in 30% sucrose for 3 days, embedded in O.C.T., and then sectioned with a cryostat.

**(B):** Experimental design for intracerebral photoconversion in KikGR transgenic mice. After EAE induction, a 405 nm wavelength fiber optic was funneled through a short bevel 18-gauge needle to intracerebrally photoconvert cells within the CNS parenchyma. The CNS was photoconverted for 5 minutes, once on Day 15 post-immunization and again on Day 16 post-immunization.

**(C):** Experimental design for visualizing CSF efflux into cribriform plate lymphatics. During peak EAE, 10 µl of Evans blue dye was injected into the cisterna magna at a rate of 2 µl/minute. Evans blue dye was able to stain and be visualized within cribriform plate lymphatics despite extensive decalcification and washing.

**(D):** Experimental design for functionally measuring CNS derived antigen drainage. EAE was induced in CNP-OP transgenic mice which express ovalbumin peptides on oligodendrocytes using the CNP promoter. Beginning on Day 7 post-immunization, these mice were intraperitoneally treated with either vehicle or 10 mg/kg of MAZ51 once per day until sacrifice. On Day 10 post-immunization, splenocytes from congenic Thy1.1, ovalbumin specific OT-I and OT-II cells labeled with CellTrace Violet were intravenously injected into recipient mice.

**
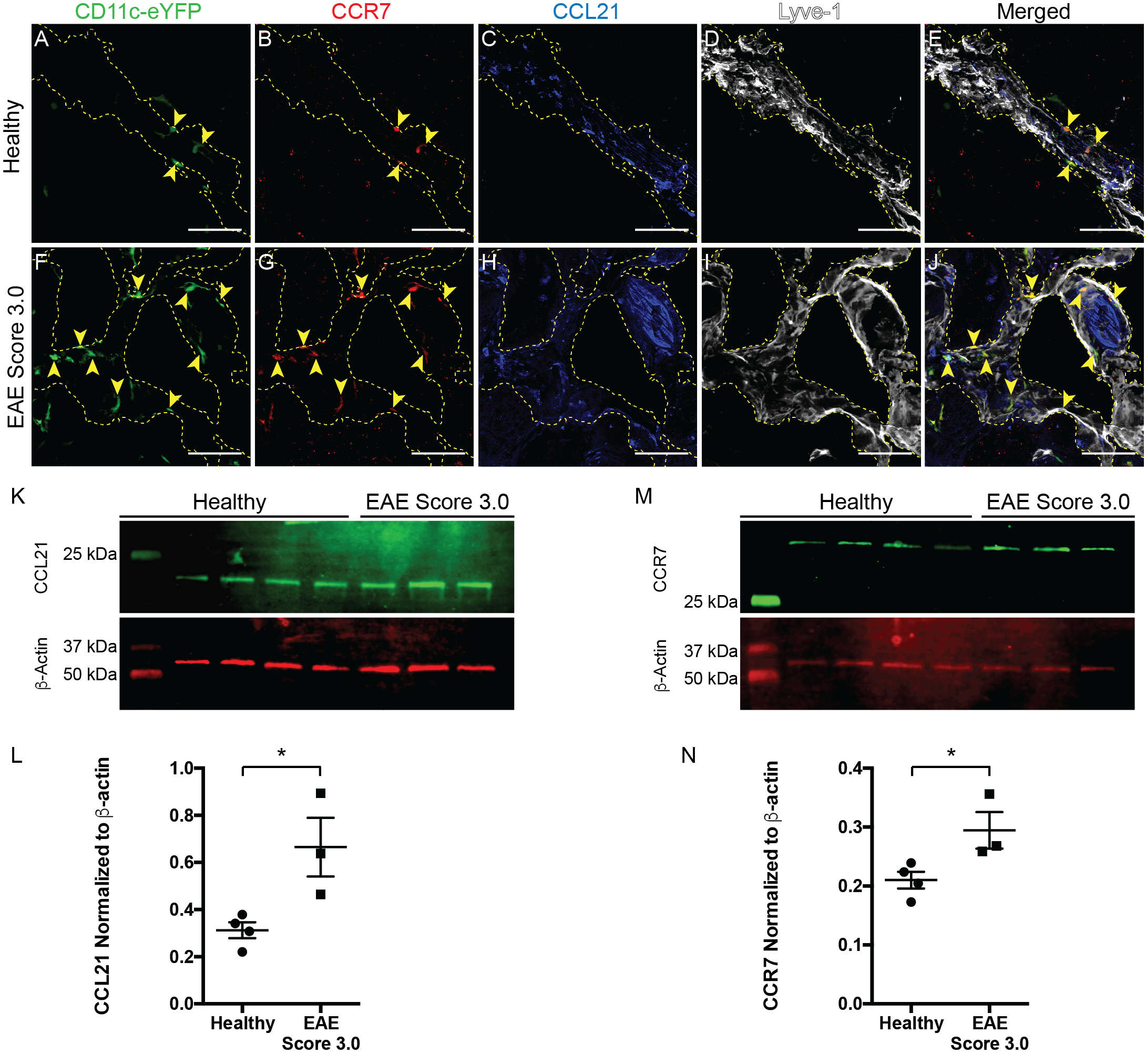
**

**Supplementary Figure 2: Cribriform plate lymphatics express CCL21 and correlate with CCR7^+^ CD11c-eYFP^+^ cell accumulation.**

**(A – E):** The whole-heads of healthy CD11c-eYFP transgenic reporter mice were coronally sectioned and immunolabeled with the migratory chemokine receptor CCR7, its ligand CCL21, and Lyve-1 to visualize CCR7^+^ CD11c-eYFP^+^ dendritic cells near and within CCL21^+^ Lyve-1^+^ lymphatic vessels near the cribriform plate. Scale bars = 100 µm.

**(F – J):** EAE was induced in CD11c-eYFP transgenic reporter mice, and on Day 18 post-immunization were harvested, the whole heads coronally sectioned, and immunolabeled with the migratory chemokine receptor CCR7, its ligand CCL21, and Lyve-1 to visualize CCR7^+^ CD11c-eYFP^+^ dendritic cells near and within CCL21^+^ Lyve-1^+^ lymphatic vessels near the cribriform plate. Scale bars = 100 µm.

**(K -L):** CNS lysates from healthy and EAE score 3.0 wild-type mice were probed for CCL21 and ß-Actin as a loading control by western blot **(K)**. Quantitation of the relative intensity of CCL21 normalized to ß-Actin revealed a significant increase in CCL21 protein expression within the CNS (*n* = 3-4 mice per group; data are represented as mean ± SEM, **p* < 0.05, unpaired Student’s t-test) **(L)**.

**(M – N):** CNS lysates from healthy and EAE score 3.0 wild-type mice were probed for CCR7 and ß-Actin as a loading control by western blot **(M)**. Quantitation of the relative intensity of CCR7 normalized to ß-Actin revealed a significant increase in CCR7 protein expression within the CNS (*n* = 3-4 mice per group; data are represented as mean ± SEM, **p* < 0.05, unpaired Student’s t-test) **(N)**.


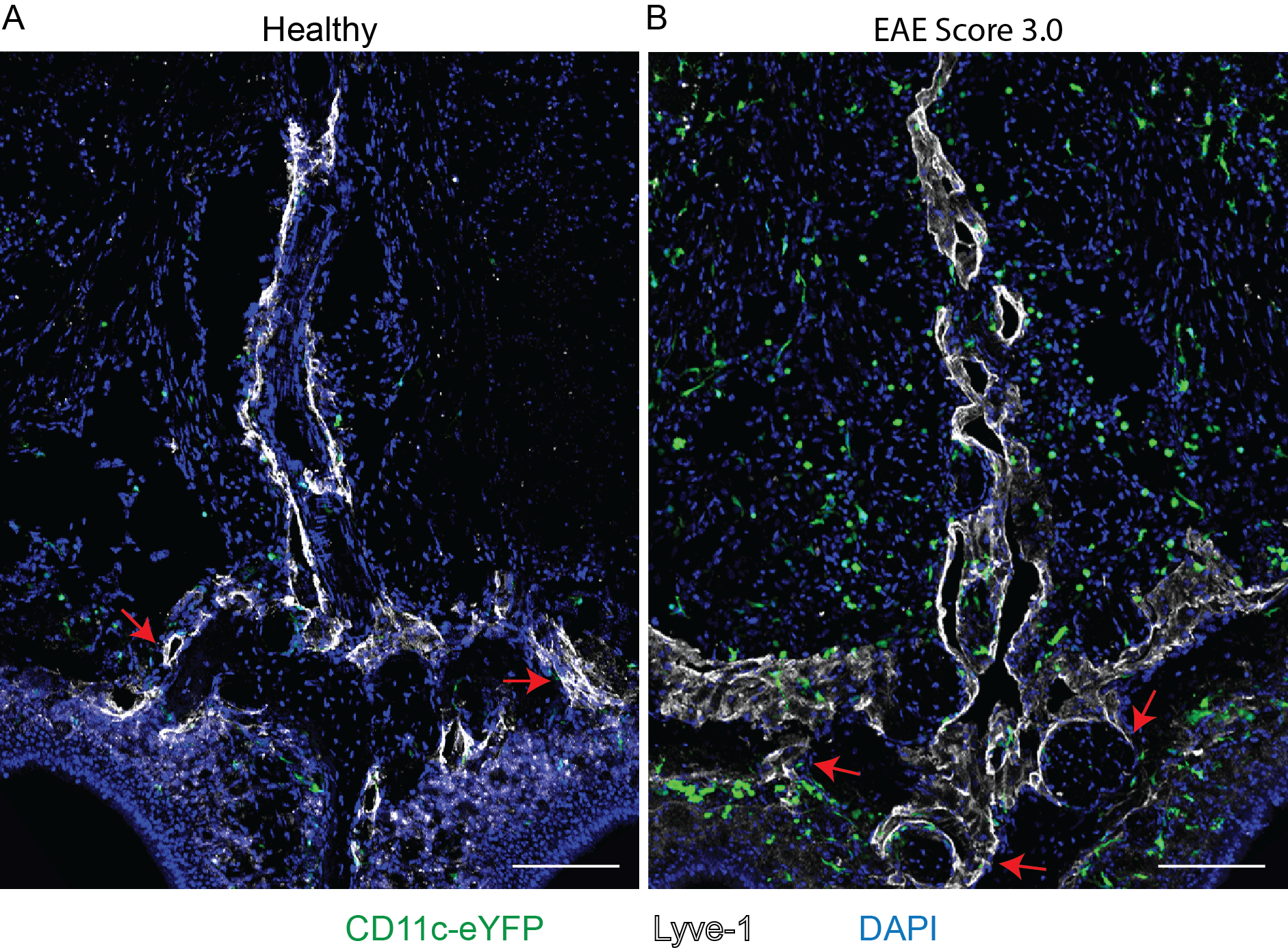


**Supplementary Figure 3: Lymphatic vessels physically cross through the cribriform plate.**

**(A):** Representative coronal section of the cribriform plate and surrounding tissues taken from a healthy CD11c-eYFP transgenic reporter mouse and immunolabeled with Lyve-1 and DAPI. Red arrows indicate lymphatic vessels passing through the cribriform plate and into the nasal mucosa. Scale bars = 100 µm.

**(B):** Representative coronal section of the cribriform plate and surrounding tissues taken from an EAE Score 3.0 CD11c-eYFP transgenic reporter mouse and immunolabeled with Lyve-1 and DAPI. Red arrows indicate lymphatic vessels passing through the cribriform plate and into the nasal mucosa. Note the increase in lymphatic vessel diameter and its expansion. Scale bars = 100 µm.


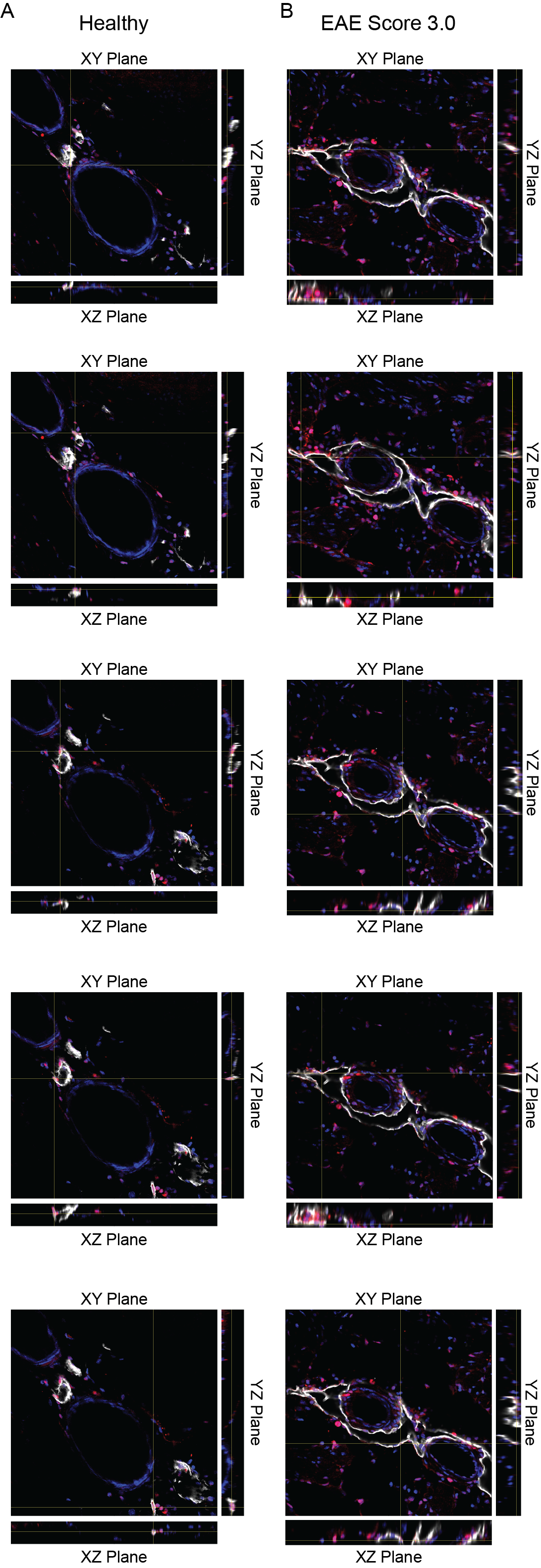


**Supplementary Figure 4: Orthogonal view of Ki67^+^ lymphatic endothelial cells.**

**(A – B):** Representative orthogonal views taken from a healthy **(A)** or EAE Score 3.0 **(B)** mice as shown in **Figure 2**. Orthogonal views show the Ki67^+^ cell of interest in the XY, XZ, and YZ planes co-localizing with Lyve-1^+^ lymphatic endothelial cells, suggesting these cells are indeed proliferating Ki67^+^ lymphatic endothelial cells.

**
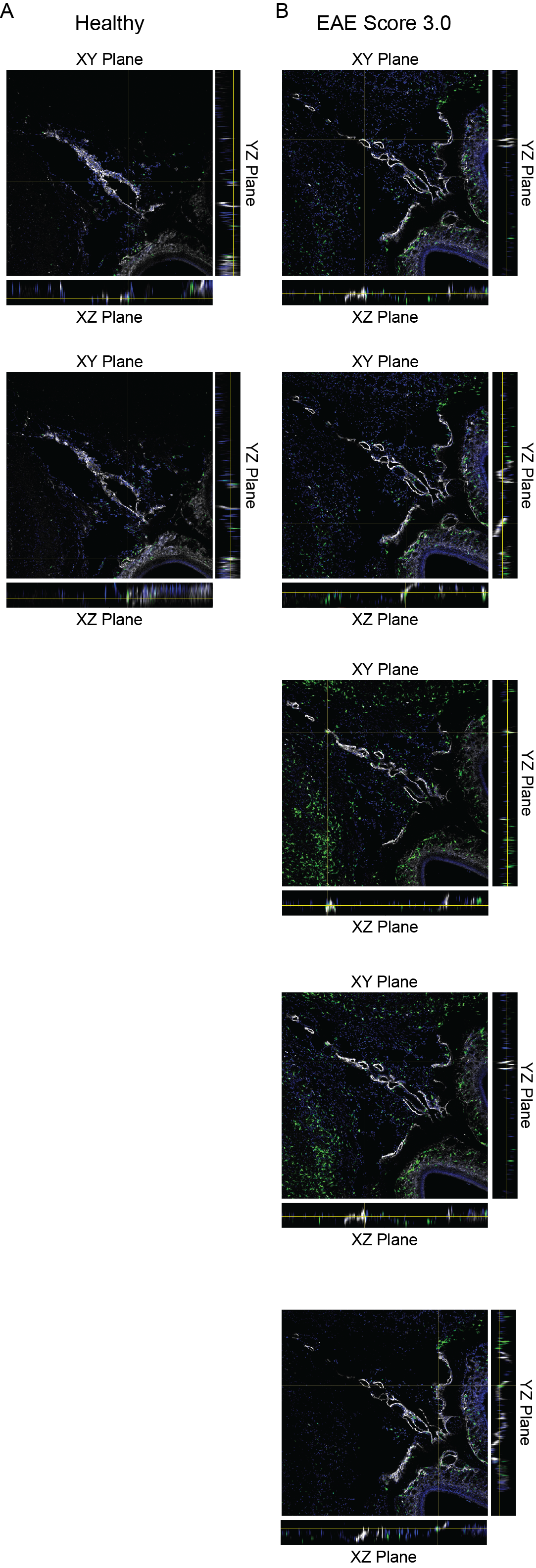
**

**Supplementary Figure 5: Orthogonal view of CD11c-eYFP^+^ cells within lymphatic vessels.**

**(A – B):** Representative orthogonal views taken from a healthy **(A)** or EAE Score 3.0 **(B)** mice as shown in **Figure 2**. Orthogonal views show the CD11c-eYFP^+^ cell of interest in the XY, XZ, and YZ planes co-localizing with Lyve-1^+^ lymphatic endothelial cells, suggesting these cells are indeed CD11c-eYFP^+^ dendritic cells migrating into lymphatic vessels near the cribriform plate.

**
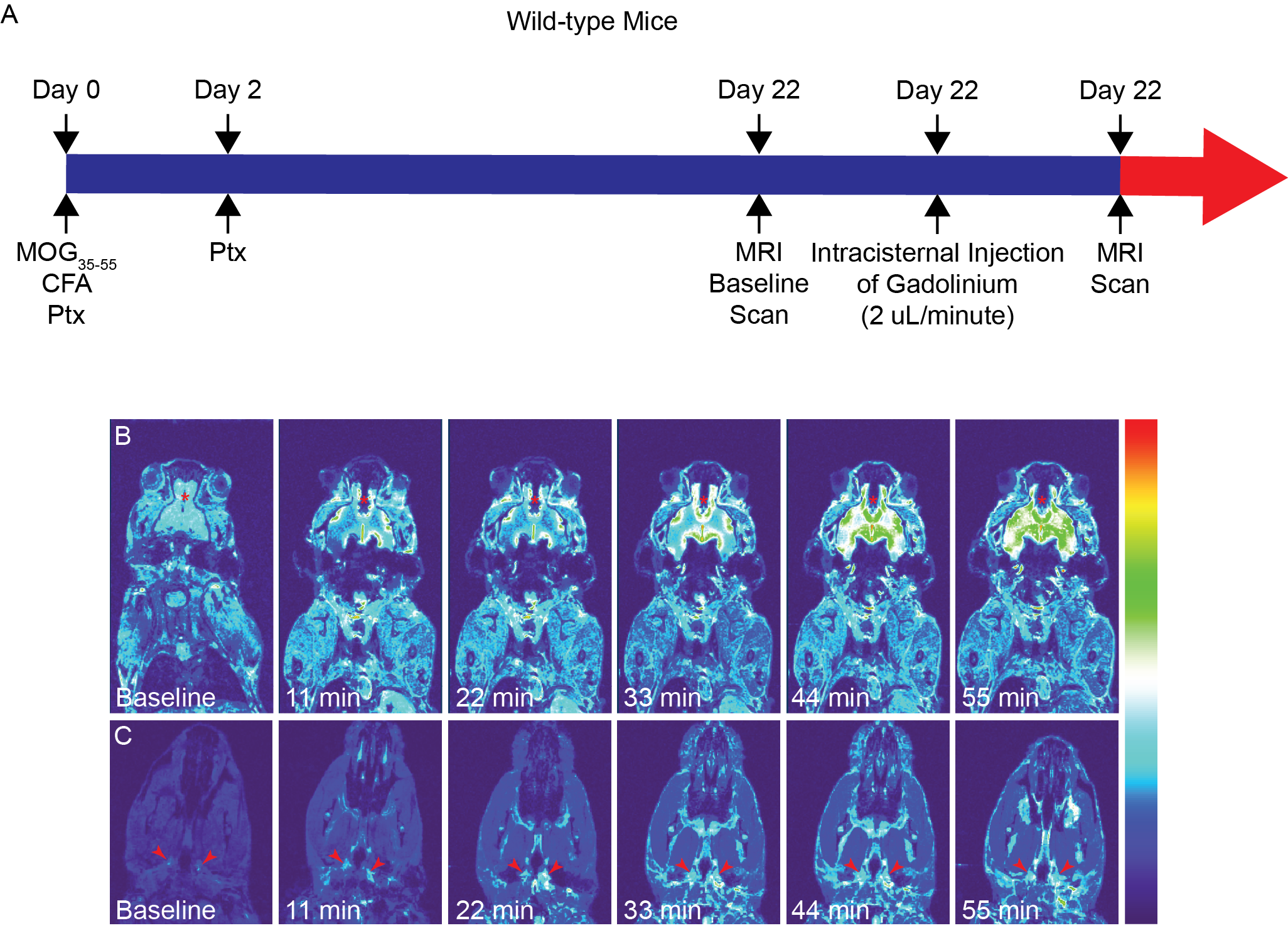
**

**Supplementary Figure 6: CSF accumulates at the base of the brain and near the cribriform plate.**

**(A**): Schematic of experimental design for dynamically visualizing CSF accumulation in an EAE Score 3.0 wild-type mouse. Baseline magnetic resonance imaging images were taken before the administration of gadolinium.

**(B):** 10 µl of Gadolinium was injected into the cisterna magna at a rate of 2 µl/minute and immediately underwent continuous imaging for approximately 55 minutes. Note the accumulation of gadolinium near the base of the brain and surrounding the middle nasal septum and cribriform plate over time. Red asterisks indicate where the cribriform plate is located. Data is representative of 5 animals.

**(C):** Magnetic resonance imaging also reveals increased drainage of gadolinium into the deep cervical lymph nodes over time. Red arrowheads indicate the location of deep cervical lymph nodes.

**
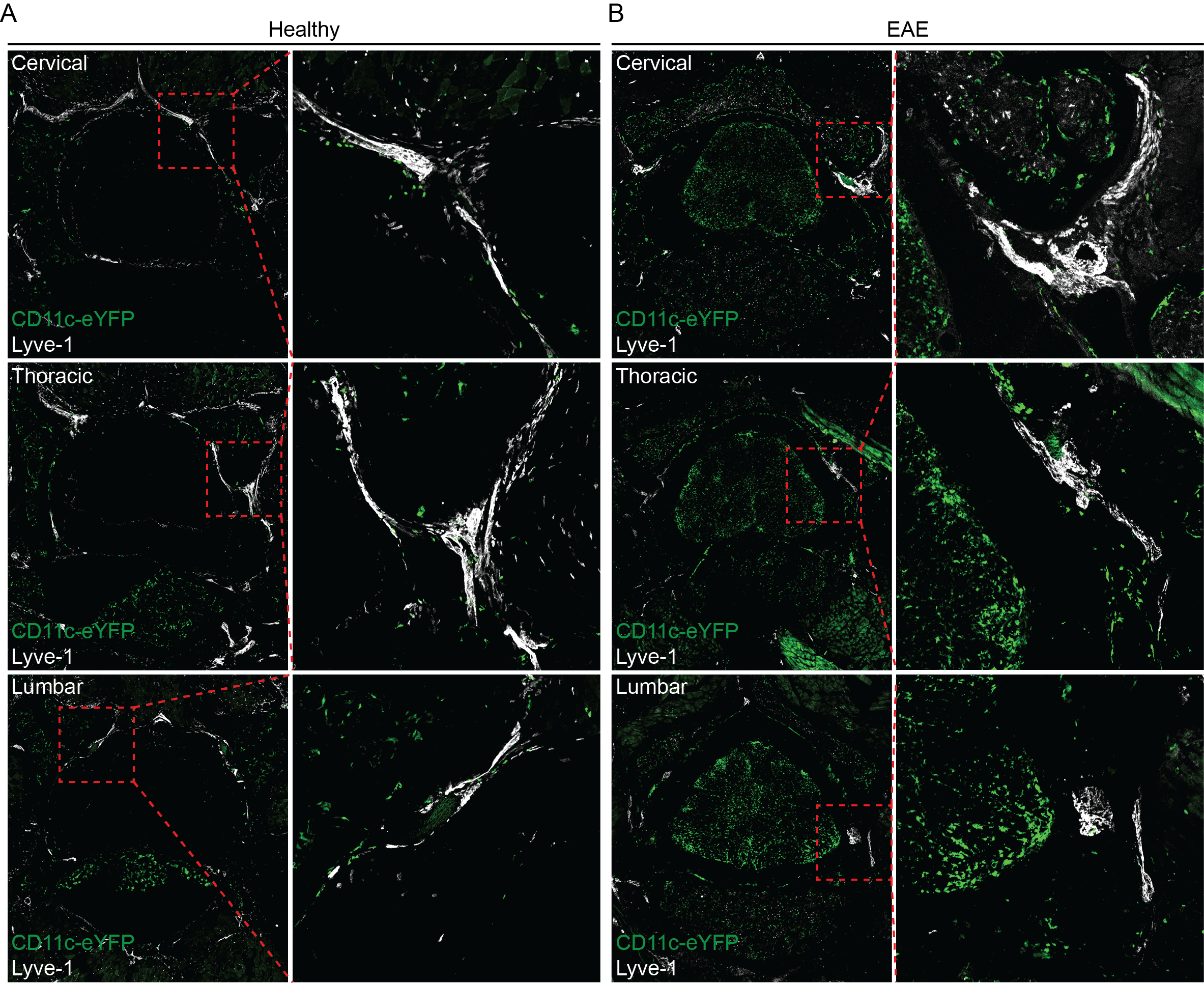
**

**Supplementary Figure 7: Neuroinflammation does not induce lymphangiogenesis in lymphatics surrounding the spinal cord.**

**(A**): The spinal columns were harvested from healthy CD11c-eYFP transgenic reporter mice and underwent decalcification. Coronal sections of the whole spinal columns immunolabeled with Lyve-1 reveal lymphatic vessels exiting the dura of the spinal cord along nerve rootlets in the cervical, thoracic, and lumbar regions of the spinal cord.

**(B**): The spinal columns were harvested from EAE Score 3.0 CD11c-eYFP transgenic reporter mice and underwent decalcification. Coronal sections of the whole spinal columns immunolabeled with Lyve-1 reveal lymphatic vessels exiting the dura of the spinal cord along nerve rootlets in the cervical, thoracic, and lumbar regions of the spinal cord. Note the lack of any macroscopic changes to lymphatic vessels between healthy and EAE mice.


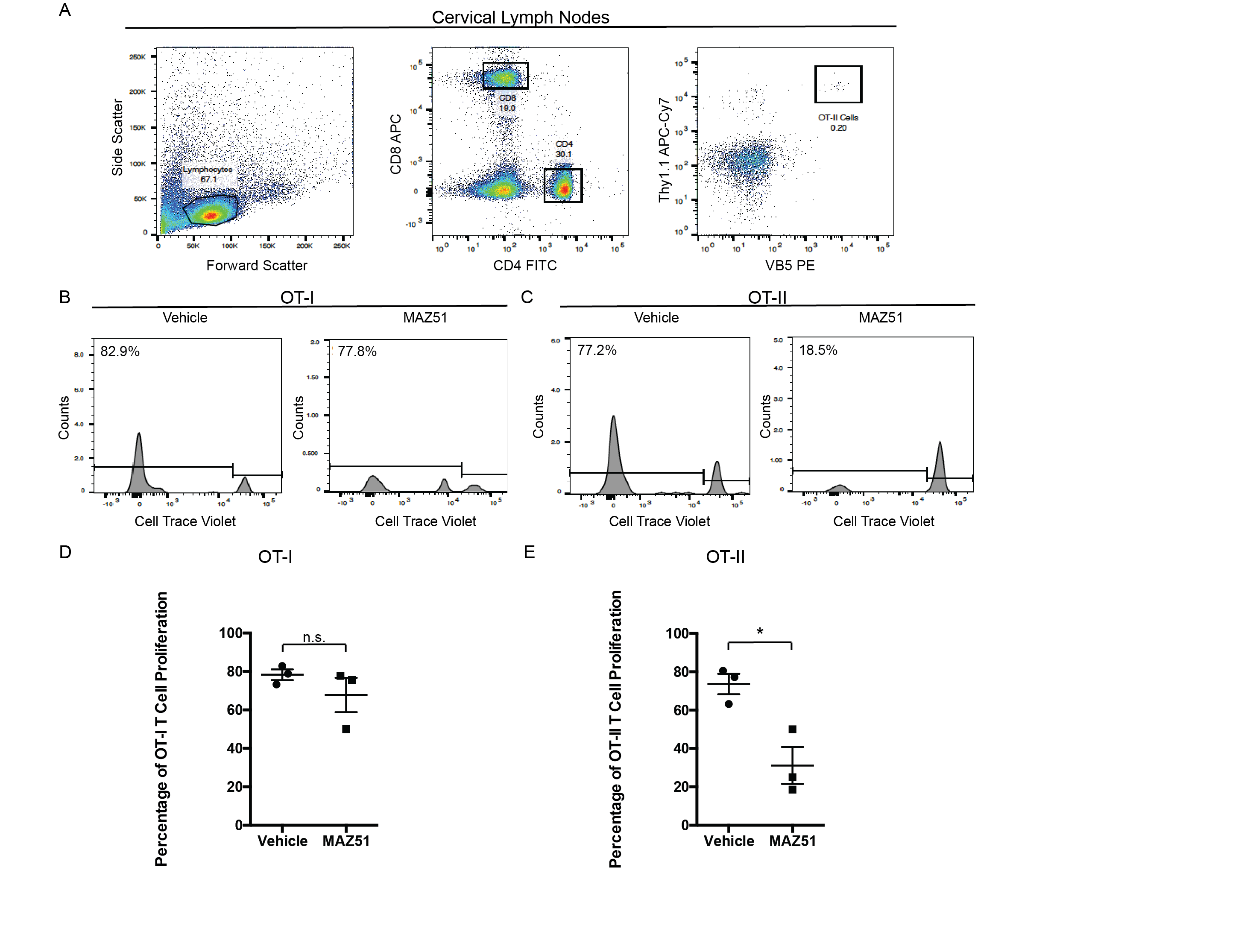
**Supplementary Figure 8: Pharmacological inhibition of lymphangiogenesis reduces CNS antigen specific CD4 T cell proliferation in Nes-OP transgenic mice.**

**(A-E):** ß-gal floxed Ovalbumin Peptides (OP) transgenic mice were crossed with Nestin-Cre transgenic mice to drive ovalbumin peptide expression on neural progenitor cells (Nes-OP mice). These mice were then induced with EAE, I.P. treated with either vehicle or the VEGFR3 tyrosine kinase inhibitor MAZ51 beginning on Day 7 post-immunization, and Ovalbumin specific CD8 (OT-I) or CD4 (OT-II) Thy1.1 congenic CellTrace Violet labeled T cells were adoptively transferred on Day 12 post-immunization. T cell proliferation was then measured by Flow Cytometry.

**(A):** Gating strategy for the adoptively transferred, CellTrace Violet labeled, Thy1.1 congenic, Vß5^+^, Ovalbumin specific T cells.

**(B-C):** Representative histograms of CellTrace Violet proliferation for OT-I **(B)** or OT-II **(C)** T cells.

**(D-E):** Quantitation of the average percent of OT-I **(D)** or OT-II **(E)** T cell proliferation in the draining lymph nodes at day 18 post-immunization (data are represented as mean ± SEM; *n* = 3 mice per group; N.S. = non-significant, **p* < 0.05, unpaired Student’s t-test)


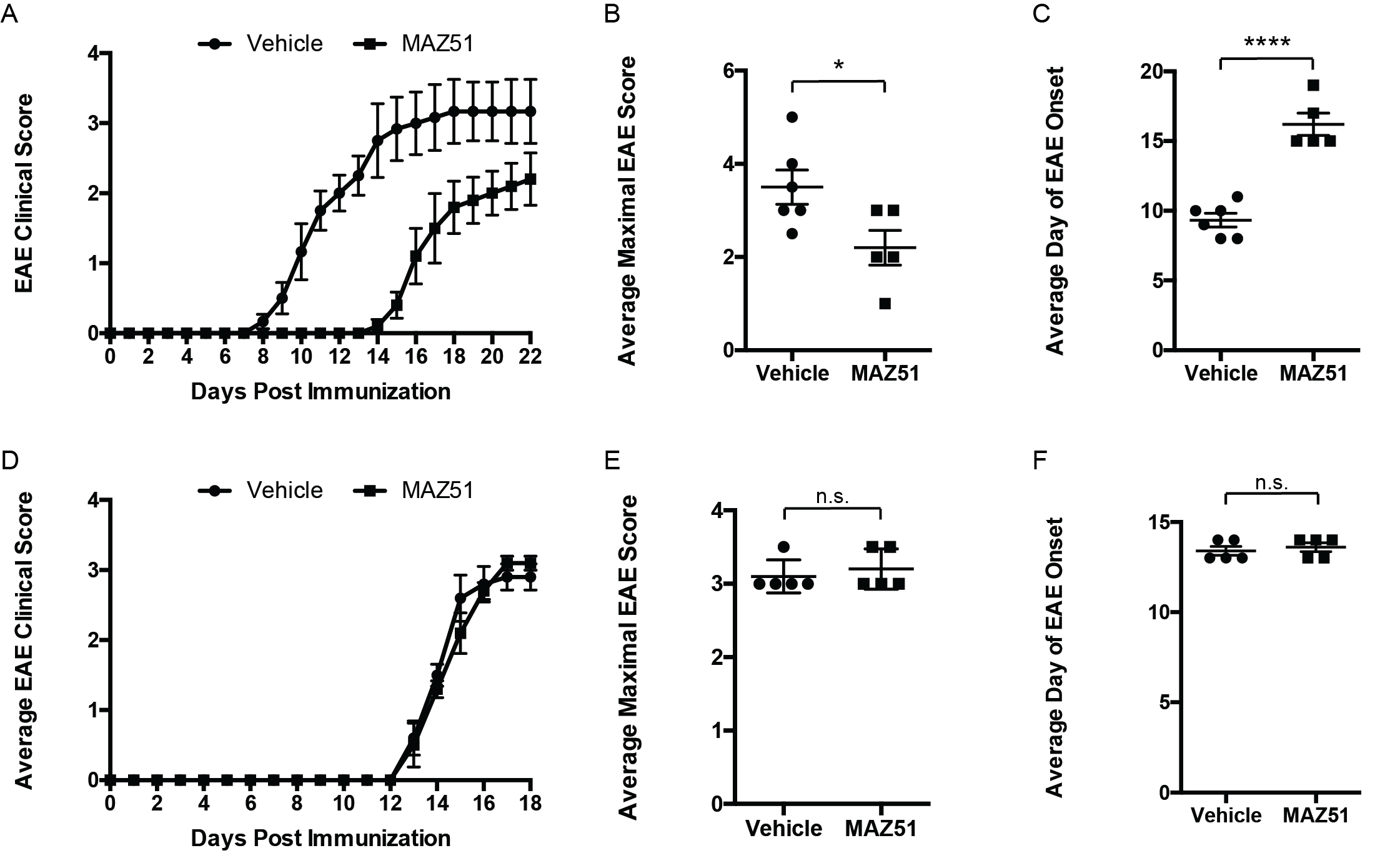


**Supplementary Figure 9: Pharmacological inhibition of VEGFR3 delays the onset and reduces EAE severity when administered prior to clinical symptoms.**

**(A – C):** EAE was induced in wild-type mice and were intraperitoneally treated with either vehicle or MAZ51 beginning on Day 7 post-immunization, once per day until sacrifice. Mice receiving I.P. MAZ51 once per day beginning on Day 7 post-immunization have reduced EAE severity **(A)**, delay in the onset of EAE **(B)** (*n* = 5-6 mice per group; data are represented as mean ± SEM, **p* < 0.05, unpaired Student’s t-test), and reduced average maximal EAE scores **(C)** (*n* = 5-6 mice per group; data are represented as mean ± SEM, **p* < 0.05, unpaired Student’s t-test).

**(D – F):** EAE was induced in wild-type mice and were intraperitoneally treated with either vehicle or MAZ51 after the onset of EAE clinical symptoms, once per day until sacrifice. Mice receiving I.P. MAZ51 have no change in EAE clinical scores when compared to vehicle treated animals **(A)**, no change in the onset of EAE **(B)** (*n* = 5-6 mice per group; data are represented as mean ± SEM, N.S. = non-significant, unpaired Student’s t-test), and no change in the average maximal EAE scores **(C)** (*n* = 5 mice per group; data are represented as mean ± SEM, n.s. = non-significant, unpaired Student’s t-test).


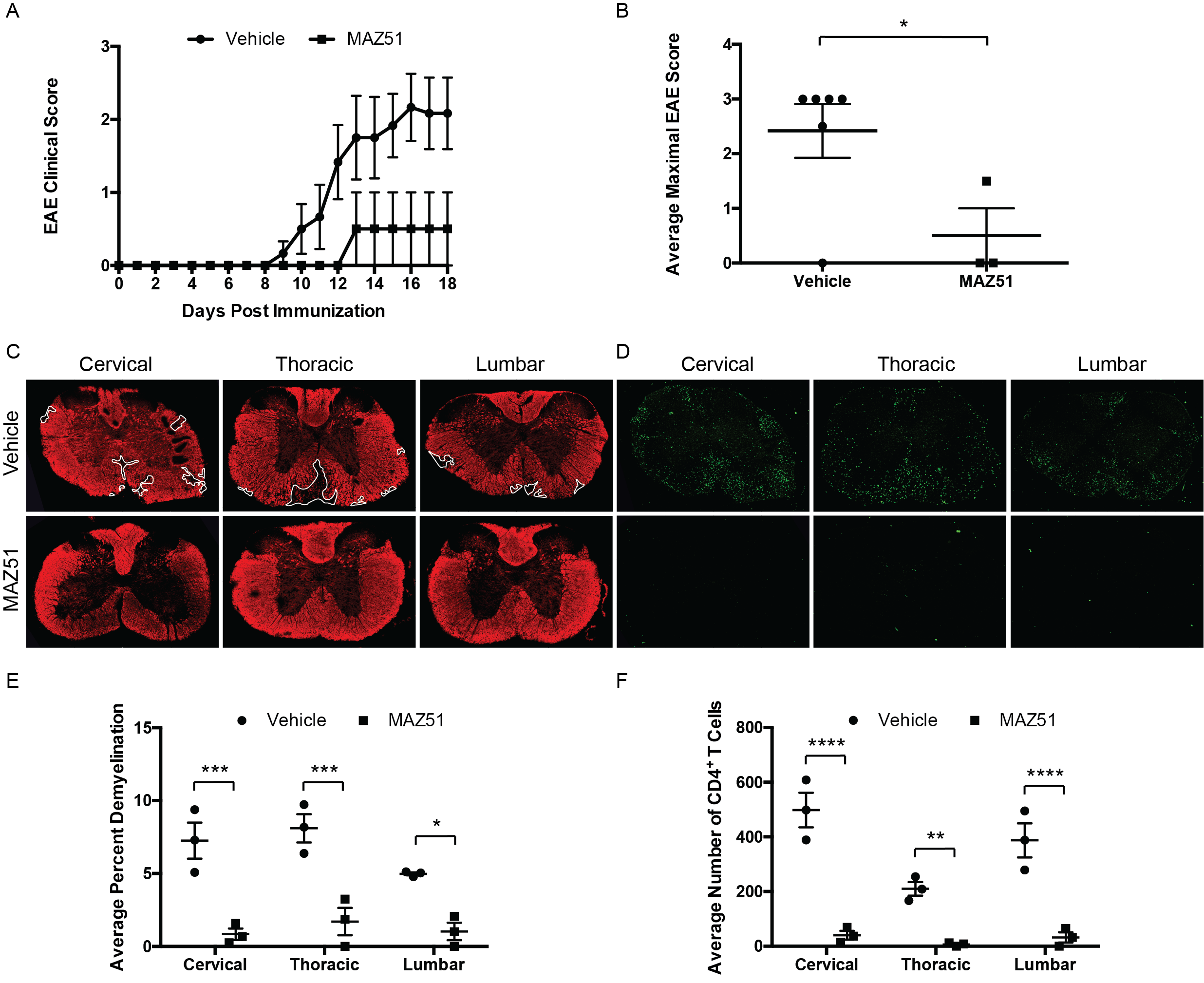


**Supplementary Figure 10: Pharmacological inhibition of VEGFR3 reduces EAE severity in Nes-OP transgenic mice.**

**(A-B):** ß-gal floxed Ovalbumin Peptides (OP) transgenic mice were crossed with a neural progenitor specific Cre mouse (Nestin-Cre) to generate Nes-OP transgenic mice. EAE was induced in Nes-OVA mice, treated with either vehicle or MAZ51, and were adoptively transferred CellTrace Violet labeled, Thy1.1 congenic, CD8 (OT-I) and CD4 (OT-II) T cells as shown in Figure 5. Mice receiving I.P. MAZ51 once per day beginning on Day 7 post-immunization have reduced EAE severity **(A)** and reduced average maximal EAE scores **(B)** (*n* = 3 – 6 mice per group; data are represented as mean ± SEM, **p* < 0.05, unpaired Student’s t-test).

**(C-D):** The spinal cords of MAZ51 and vehicle treated animals were immunolabeled with Fluoromyelin to visualize myelin within the spinal cord **(C)**. MAZ51 treated mice have significantly reduced percent demyelination when compared to vehicle treated controls **(D)** (*n* = 3 mice per group; data are represented as mean ± SEM, **p* < 0.05, ****p* < 0.001, two-way ANOVA with Sidaks multiple comparisons test).

**(E-F):** The spinal cords of MAZ51 and vehicle treated animals were immunolabeled with CD4 to visualize CD4 T cell infiltration into the spinal cord **(E)**. MAZ51 treated mice have significantly reduced CD4 T cell infiltration into the spinal cord when compared to vehicle treated controls **(F)** (*n* = 3 mice per group; data are represented as mean ± SEM, ***p* < 0.01, ****p < 0.0001, two-way ANOVA with Sidaks multiple comparisons test).


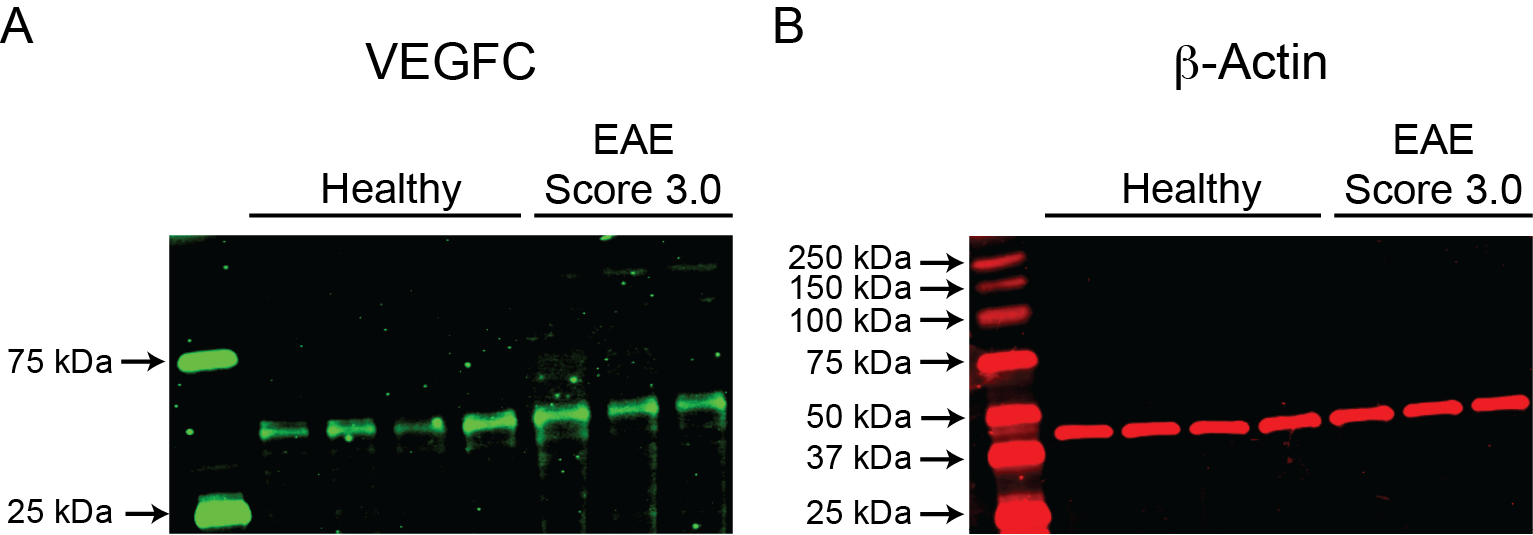


**Supplementary Figure 11: Uncropped Western Blots from Figure 4**

**(A):** Uncropped Western blot from Figure 4 for VEGFC.

**(B):** Uncropped Western blot from Figure 4 for ß-Actin.

| **Reagent** | **Source** | **Reference Number** |
| --- | --- | --- |
| **Antibodies** | | |
| Rat ∝-Lyve-1 Conjugated to eFluor-660 | eBioscience | 50-0443-82 |
| Rat ∝-Lyve-1 Conjugated to eFluor-570 | eBioscience | 41-0443-82 |
| Rat ∝-Podoplanin Conjugated to eFluor-488 | eBioscience | 53-5381-80 |
| Goat ∝-VEGFR3 | R&D Systems | AF743-SP |
| Goat ∝-CCL21 | R&D Systems | AF457-SP |
| Rat ∝-CCR7 Conjugated to PE | BD Pharmingen | 560682 |
| Rabbit ∝-CCR7 | Abcam | ab32527 |
| Rat ∝-CD11b Conjugated to PE | BD Pharmingen | 553311 |
| Rabbit ∝-VEGFC | Abcam | ab83905 |
| Fluoromyelin Red Fluorescent Myelin Stain | Thermo Fisher Scientific | F34652 |
| Rat ∝-CD4 Conjugated to FITC | BD Pharmingen | 553047 |
| Rat ∝-CD4 Conjugated to Alexa Fluor-647 | BD Pharmingen | 557681 |
| Rat ∝-CD8a Conjugated to FITC | BD Pharmingen | 553031 |
| Rat ∝-V𝛃 5.1, 5.2 T-Cell Receptor Conjugated to PE | BD Pharmingen | 553190 |
| Rat ∝-CD90.1 Conjugated to APC-Cy7 | BD Pharmingen | 561401 |
| Chicken ∝-𝛽-actin | Abcam | ab13822 |
| Donkey ∝-Goat Alexa Fluor 488 | Thermo Fisher Scientific | A11055 |
| Donkey ∝-Goat Alexa Fluor 405 | Abcam | ab175664 |
| Donkey ∝-Rabbit Alexa Fluor 405 | Abcam | ab175649 |
| IRDye 800CW Donkey Anti-Rabbit IgG (H+L) | Li-Cor | 926-32213 |
| IRDye 800CW Donkey Anti-Goat IgG (H+L | Li-Cor | 925-32214 |
| IRDye 800CW Donkey Anti-Goat IgG (H+L) | Li-Cor | 926-68028 |
| **Chemicals** | | |
| VEGFR3 Kinase Inhibitor, MAZ51 | EMD Millipore | 676492-10MG |
| CellTrace Violet Proliferation Kit | Thermo Fisher Scientific | C34557 |
| Rat/Mouse Myelin Oligodendrocyte Glycoprotein 35-55 | Genemed Synthesis | MOG3555-P-5 |
| Freund's Adjuvant, Complete | Sigma-Aldrich | F5881-6X10ML |
| Pertussis Toxin | Thermo Fisher Scientific | NC9675592 |
| Dimethyl Sulfoxide | Fisher Scientific | D128-500 |
| Collagenase/Dispase | Sigma-Aldrich | 1026938001 |
| DNAse I | Thermo Fisher Scientific | AM2222 |
| Ethylenediaminetetraacetic Acid | Fisher Scientific | BP118-500 |
| ProLong Gold Antifade Mount with DAPI | Thermo Fisher Scientific | P36935 |
| Percoll | GE Healthcare Life Sciences | 17-0891-01 |
| HBSS | Corning | 21-021-CV |
| RPMI-1640 | Corning | 10-040-CV |
| Protease and Phosphatase Inhibitor Cocktail | Thermo Fisher Scientific | 78440 |
| **Mice** | | |
| C57BL/6J (H2b) Wild-type | Jackson Labs | 000664 |
| B6.Cg-Tg(Itgax-Venus)1Mnz/J (CD11c-eYFP) | Dr. Michel C. Nussenzweig at Rockefeller University, NY | |
| C57BL/6-Tg(Prox1-tdTomato)12Nrud/J | Jackson Labs | 008829 |
| B6.PL-Thy1^a^/CyJ | Jackson Labs | 022766 |
| B6.Cg-Tg(Nes-Cre)1/Kln/J | Jackson Labs | 000406 |
| C57BL/6-Tg(TcraTcrb)1100Mjb/J | Jackson Labs | 000771 |
| B6.Cg-Tg(TcraTcrb)425Cbn/J | Jackson Labs | 004194 |
| C57BL/6-Tg(TcraTcrb)1100Mjb/J | Jackson Labs | 003831 |
| pZ/EG-OP OVA_257-264_-OVA_323-339_ Floxed Mouse | Generated by our lab. | |
| Tg(CAG-KikGR)33HADj/J | Jackson Labs | 013753 |
| **Software** | | |
| GraphPad Prism 6 | GraphPad | https://www.graphpad.com/scientific-software/prism/ |
| FIJI | ImageJ | https://fiji.sc |
| FlowJo | FlowJo, LLC (TreeStar) | https://www.flowjo.com/solutions/flowjo |

**Supplementary Table 1: List of Key Reagents**
