# Supplementary material for: Neuroinflammation-induced lymphangiogenesis near the cribriform plate contributes to drainage of CNS-derived antigens and immune cells
Source: Nat Commun. 2019 Jan 16;10:229. doi: 10.1038/s41467-018-08163-0 (PMC6335416; doi:10.1038/s41467-018-08163-0)
Supplement: Supplementary file 9 — Reporting Summary [file 41467_2018_8163_MOESM9_ESM.pdf]

nature research

Corresponding author(s): *Double-blind peer review submissions: write DBPR and your manuscript number here instead of author names.*

Reporting Summary

Nature Research wishes to improve the reproducibility of the work that we publish. This form provides structure for consistency and transparency in reporting. For further information on Nature Research policies, see Authors & Referees and the Editorial Policy Checklist.

Statistical parameters

When statistical analyses are reported, confirm that the following items are present in the relevant location (e.g. figure legend, table legend, main text, or Methods section).

n/a	Confirmed	
☐	☒	The exact sample size (*n*) for each experimental group/condition, given as a discrete number and unit of measurement
☐	☒	An indication of whether measurements were taken from distinct samples or whether the same sample was measured repeatedly
☐	☒	The statistical test(s) used AND whether they are one- or two-sided<br>*Only common tests should be described solely by name; describe more complex techniques in the Methods section.*
☐	☒	A description of all covariates tested
☐	☒	A description of any assumptions or corrections, such as tests of normality and adjustment for multiple comparisons
☐	☒	A full description of the statistics including central tendency (e.g. means) or other basic estimates (e.g. regression coefficient) AND variation (e.g. standard deviation) or associated estimates of uncertainty (e.g. confidence intervals)
☐	☒	For null hypothesis testing, the test statistic (e.g. *F*, *t*, *r*) with confidence intervals, effect sizes, degrees of freedom and *P* value noted<br>*Give P values as exact values whenever suitable.*
☒	☐	For Bayesian analysis, information on the choice of priors and Markov chain Monte Carlo settings
☒	☐	For hierarchical and complex designs, identification of the appropriate level for tests and full reporting of outcomes
☒	☐	Estimates of effect sizes (e.g. Cohen's *d*, Pearson's *r*), indicating how they were calculated
☐	☒	Clearly defined error bars<br>*State explicitly what error bars represent (e.g. SD, SE, CI)*

Our web collection on statistics for biologists may be useful.

Software and code

Policy information about availability of computer code

Data collection	The following software were used for data collection:<br>-FV10-ASW v4.2<br>-Image Studio Software v5.2.5<br>-BD FACSDiva v6.1.2
Data analysis	The following software were used for data analysis:<br>-FIJI image processing software (NIH)<br>-FlowJo version (v) 10 (TreeStar)<br>-GraphPad Prism v6 (GraphPad Software Inc)<br>-Excel 16.15<br>-Image Studio Software v5.2.5

For manuscripts utilizing custom algorithms or software that are central to the research but not yet described in published literature, software must be made available to editors/reviewers upon request. We strongly encourage code deposition in a community repository (e.g. GitHub). See the Nature Research guidelines for submitting code & software for further information.

Data

Policy information about availability of data

All manuscripts must include a data availability statement. This statement should provide the following information, where applicable:

- Accession codes, unique identifiers, or web links for publicly available datasets
- A list of figures that have associated raw data
- A description of any restrictions on data availability

All data supporting the findings of this study are available from the corresponding authors upon reasonable request.

Field-specific reporting

Please select the best fit for your research. If you are not sure, read the appropriate sections before making your selection.

☒ Life sciences ☐ Behavioural & social sciences ☐ Ecological, evolutionary & environmental sciences

For a reference copy of the document with all sections, see nature.com/authors/policies/ReportingSummary-flat.pdf

Life sciences study design

All studies must disclose on these points even when the disclosure is negative.

Sample size	Sample size was chosen in accordance with similar published experiments by other independent groups as well as our lab.
Data exclusions	No data were excluded in our experiments.
Replication	All experiments were performed using multiple animals for each condition. Any experiments that were not replicated were validated using an alternative system. For example, Ki67+ lymphatic endothelial cells and CD11c-eYFP+ cells were quantified using both Z-stack maximum intensity projections as well as orthogonal views, the reduction in EAE severity by VEGFR3 inhibition in CNP-OP mice were validated using Nes-OP transgenic mice, etc.
Randomization	All animals were randomly assigned to experimental groups.
Blinding	Experimental groups were blinded for all analysis.

Reporting for specific materials, systems and methods

Materials & experimental systems

n/a	Involved in the study	
☒	☐	Unique biological materials
☐	☒	Antibodies
☒	☐	Eukaryotic cell lines
☒	☐	Palaeontology
☐	☒	Animals and other organisms
☒	☐	Human research participants

Methods

n/a	Involved in the study	
☒	☐	ChIP-seq
☐	☒	Flow cytometry
☐	☒	MRI-based neuroimaging

Antibodies

Antibodies used

Primary antibodies used for flow cytometry were used as follows: rat anti-CD4-FITC-conjugated (1:200; 553047; BD Pharmingen), rat anti-CD4-AF647-conjugated (1:200; 557681; BD Pharmingen), rat anti-CD8-FITC-conjugated (1:200; 553031; BD Pharmingen), rat anti-CD8-APC-conjugated (1:200; 553035; BD Biosciences), rat anti-Vß5.1, 5.2-PE-conjugated (1:200; 553190; BD Pharmingen), rat anti-CD90.1-PE-conjugated (1:200; 202523; Biolegend), and rat anti-CD90.1-APC-Cy7-conjugated (1:200; 561401; BD Biosciences).

Primary antibodies used for immunohistochemistry were used as follows: rat anti-Lyve-1-eFluor-570-conjugated (1:100; 41-0443-82; eBioscience), rat anti-Lyve-1-eFluor-660 conjugated (1:500; 50-0443-82; eBioscience), rat anti-Podoplanin-eFluo488 conjugated (1:100; 53-5381-80; eBioscience), goat anti-VEGFR3-unconjugated (1:100; AF743-SP; R&D Systems), rat anti-CD11b-PE conjugated (1:100; 553311; BD Pharmingen), rabbit anti-VEGFC-unconjugated (1:100; ab83905; Abcam), goat anti-CCL21-unconjugated (AF457-SP; R&D Systems), rat anti-CCR7-PE conjugated (560682; BD Pharmingen), rat anti-CD4-FITC conjugated

(1:100; 553047; BD Pharmingen), and Fluoromyelin Red Fluorescent Myelin Stain (1:1000; F34652; Thermo Fisher Scientific). The appropriate secondary antibodies were used as follows: donkey anti-goat-AF488-conjugated (1:500; A11055; Thermo Fisher Scientific), donkey anti-goat-AF405 (1:500; ab175664; Abcam), and donkey anti-rabbit-AF405 (1:500; ab175649; Abcam).

Primary antibodies used for western blot were used as follows: goat anti-CCL21-unconjugated (1:100; AF457-SP; R&D Systems), rabbit anti-CCR7-unconjugated (1:100; ab32527; Abcam), rabbit anti-VEGFC-unconjugated (1:100; ab83905; Abcam), and chicken anti-ß-actin-unconjugated (1:1000; ab13822; Abcam). The appropriate secondary antibodies were used as follows: donkey anti-rabbit IgG-IRDye 800CW (1:2000; 926-32213; Li-Cor), donkey anti-goat IgG-IRDye 800CW (1:2000; 925-32214; Li-Cor), and donkey anti-chicken IgG-IRDye 680LT (1:2000; 926-68028; Li-Cor).

Validation

All antibodies were validated for use in mice and their applications (immunohistochemistry, flow cytometry, and western blotting) were validated by the corresponding manufacturer.

Animals and other organisms

Policy information about studies involving animals; ARRIVE guidelines recommended for reporting animal research

Laboratory animals

All animals used were in C57BL/6J background between 8-12 weeks old. All animals used for EAE experiments and their appropriate healthy controls were female.
-Wild-type
-C57BL/6-Tg(Prox1-tdTomato)12Nrud/J
-B6.PL-Thy1a/CyJ (Thy1.1 congenic mice)
-C57BL/6-Tg(TcraTcrb)1100Mjb/J (ovalbumin specific CD8 T cell or OT-I mice)
-B6.Cg-Tg(TcraTcrb)425Cbn/J (ovalbumin specific CD4 T cell or OT-II mice)
-B6.Cg-Tg(Nes-Cre)1Kln/J, and Tg(CAG-KikGR)33Hadj/J
-B6.Cg-Tg(Itgax-Venus)1Mnz/J (CD11c-eYFP mice)
-Cnp-Cre
-pZ/EG-OP OVA257-264-OVA323-339 (Ovalbumin floxed mice)

Wild animals

Not Applicable

Field-collected samples

Not Applicable

Flow Cytometry

Plots

Confirm that:

☒ The axis labels state the marker and fluorochrome used (e.g. CD4-FITC).

☒ The axis scales are clearly visible. Include numbers along axes only for bottom left plot of group (a 'group' is an analysis of identical markers).

☒ All plots are contour plots with outliers or pseudocolor plots.

☒ A numerical value for number of cells or percentage (with statistics) is provided.

Methodology

Sample preparation

Mice were perfused with 0.1M PBS (10 mL), and the cervical and deep cervical lymph nodes were collected and placed in a 70 micron mesh strainer within a small Petri dish containing 10 mL of RPMI. The back of a syringe plunger was used to push the draining lymph nodes through the strainer to generate a single cell suspension. The cells in RPMI were collected, spun down at 1258 RCF at 4 degrees celsius for 7 minutes and resuspended in ice-cold 1 mL of FACS buffer (pH 7.4, 0.1M PBS, 1mM EDTA, 1% BSA), and counted using an automated Cell Counter. Cells were then washed two more times with ice-cold FACS buffer and stained using the antibodies described above at 4 degrees celsius for 30 minutes. The cells were then washed three more times with ice-cold FACS buffer and fixed in 4% PFA in 0.1M PBS. Data was collected using an LSRII (BD Biosciences) and analyzed using FlowJo software (TreeStar). Gating was done as shown in the Figures, and data processing was done with Excel and statistics performed using GraphPad Prism. Individual numerical values for percentages can be visualized in the quantitation graphs with statistics.

Instrument

LSR II (BD Biosciences)

Software

FlowJo (TreeStar)

Cell population abundance

No post-sort samples were used for any experiments.

Gating strategy

For Figure 6, lymphocytes were gated using SSC-A and FSC-A, and the adoptively transfered OT-I T cells were gated using Thy1.1-PE and CD8-PE-Cy7 or the adoptively transfered OT-II T cells were gated using Thy1.1-PE and CD4-FITC. Cell trace violet dilution histograms are then represented from these gatings.

☒ Tick this box to confirm that a figure exemplifying the gating strategy is provided in the Supplementary Information.

Magnetic resonance imaging

Experimental design

Design type	Resting State
Design specifications	Baseline images were taken under anesthesia (scan time was approximately 11 minutes). Mice were then administered 10 uL of Gadolinium into the cisterna magna at a rate of 2 uL/minute under anesthesia, and underwent 5 sequential 3D T1-weighted scans to visualize Gadolinium distribution for a total time of approximately 55 minutes (approximately 11 minutes per scan).
Behavioral performance measures	Not Applicable

Acquisition

Imaging type(s)	Isotropic 3D T1-weighted scans
Field strength	4.7
Sequence & imaging parameters	TR = 9.3 ms; TE = 4.7 ms; Flip Angle = 20 degrees; Field of View = 40x20x20 mm; Resolution = 256x128x128; Averages = 4; Voxel Size is approximately equal to 156 microns cubed. These resulted in a time scan of approximately 11 minutes.
Area of acquisition	The ROI selected included the whole-head and neck so that the cribriform plate, subarachnoid space, the brain, and the deep cervical lymph nodes could be visualized.

Diffusion MRI ☐ Used ☒ Not used

Preprocessing

Preprocessing software	Not Applicable
Normalization	Not Applicable
Normalization template	Not Applicable
Noise and artifact removal	Not Applicable
Volume censoring	Not Applicable

Statistical modeling & inference

Model type and settings	No statistical modeling was done.
Effect(s) tested	No statistical modeling was done.

Specify type of analysis: ☐ Whole brain ☒ ROI-based ☐ Both

Anatomical location(s)	ROI were selected to encompass the whole-head and neck so that the cribriform plate, subarachnoid space, the brain, and the deep cervical lymph nodes could be visualized.
Statistic type for inference (See Eklund et al. 2016)	No statistical modeling was done.
Correction	No statistical modeling was done.

Models & analysis

n/a	Involved in the study
☒	☐ Functional and/or effective connectivity
☒	☐ Graph analysis
☒	☐ Multivariate modeling or predictive analysis
